# A Comprehensive Review of Artificial Intelligence Based Algorithms Regarding Temporomandibular Joint Related Diseases

**DOI:** 10.3390/diagnostics13162700

**Published:** 2023-08-18

**Authors:** Sifa Ozsari, Mehmet Serdar Güzel, Dilek Yılmaz, Kıvanç Kamburoğlu

**Affiliations:** 1 Department of Computer Engineering, Ankara University, 06830 Ankara, Turkey; serdarmg@gmail.com; 2 Faculty of Dentistry, Baskent University, 06490 Ankara, Turkey; dtdilek@hotmail.com; 3 Department of Dentomaxillofacial Radiology, Ankara University, 06560 Ankara, Turkey; dtkivo@yahoo.com

**Keywords:** temporomandibular joint, temporomandibular joint diseases, artificial intelligence, machine learning, deep learning

## Abstract

Today, with rapid advances in technology, computer-based studies and Artificial Intelligence (AI) approaches are finding their place in every field, especially in the medical sector, where they attract great attention. The Temporomandibular Joint (TMJ) stands as the most intricate joint within the human body, and diseases related to this joint are quite common. In this paper, we reviewed studies that utilize AI-based algorithms and computer-aided programs for investigating TMJ and TMJ-related diseases. We conducted a literature search on Google Scholar, Web of Science, and PubMed without any time constraints and exclusively selected English articles. Moreover, we examined the references to papers directly related to the topic matter. As a consequence of the survey, a total of 66 articles within the defined scope were assessed. These selected papers were distributed across various areas, with 11 focusing on segmentation, 3 on Juvenile Idiopathic Arthritis (JIA), 10 on TMJ Osteoarthritis (OA), 21 on Temporomandibular Joint Disorders (TMD), 6 on decision support systems, 10 reviews, and 5 on sound studies. The observed trend indicates a growing interest in artificial intelligence algorithms, suggesting that the number of studies in this field will likely continue to expand in the future.

## 1. Introduction

In recent years, technology has experienced rapid advancements, and its widespread application now touches almost every domain. The ability to perform numerous tasks online has significantly increased the amount of data being generated daily. To efficiently process this data, make automated inferences, and minimize reliance on human intervention, the adoption of artificial intelligence-based approaches becomes unavoidable.

AI can be defined as the process of transferring human intelligence to computers, while ML, a sub-field of AI, involves making inferences by learning from data. In recent years, Deep Learning (DL) approaches, a sub-field of Machine Learning (ML), have demonstrated remarkable effectiveness in various domains, including image processing, object detection, and classification. These DL techniques have also been applied in dentistry, as evidenced by studies such as [1,2,3,4,5,6]. The primary reason why deep learning architectures receive significant attention is their ability to automatically learn from diverse types of data, including images, signals, videos, and texts.

**Figure 1:**
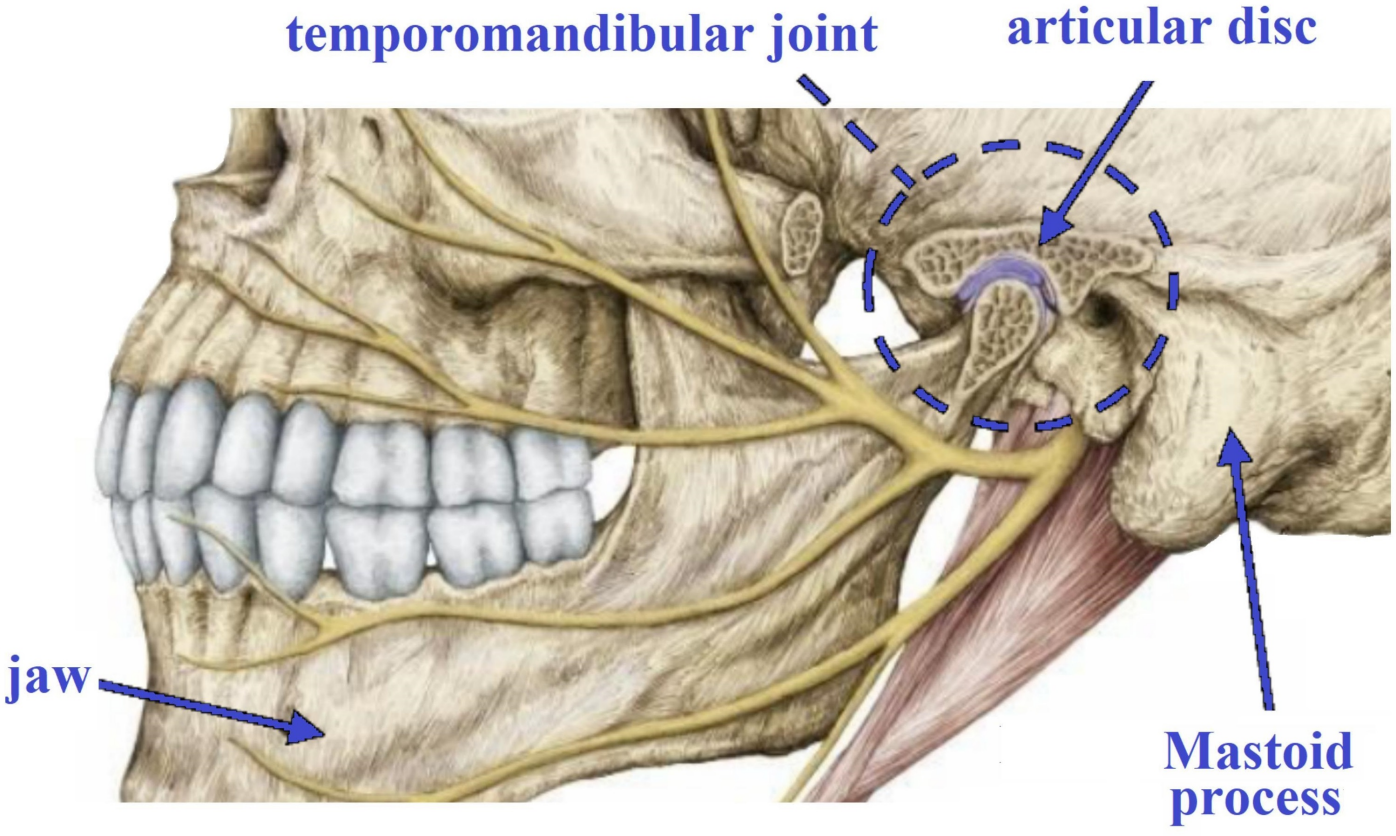
Anatomy of TMJ [10].

The ML and DL methodologies utilized in the research we examined were not explained in detail. For a more comprehensive understanding of these methods, we included two publications in the references [7,8], which provide general information about ML and DL algorithms. Researchers can refer to these and similar studies to obtain more in-depth information about the methodologies employed. The temporomandibular joint binds the mandible or lower jaw to the skull and is a complicated, delicate, and mobile joint [9]. The TMJ controls the movement of the jaw. The anatomy of the TMJ can be observed in Figure 1. Temporomandibular joint diseases, often related to muscles, are quite common. They present with various symptoms, including face, head, or jaw pain, and joint-induced sounds.

**Figure 2:**
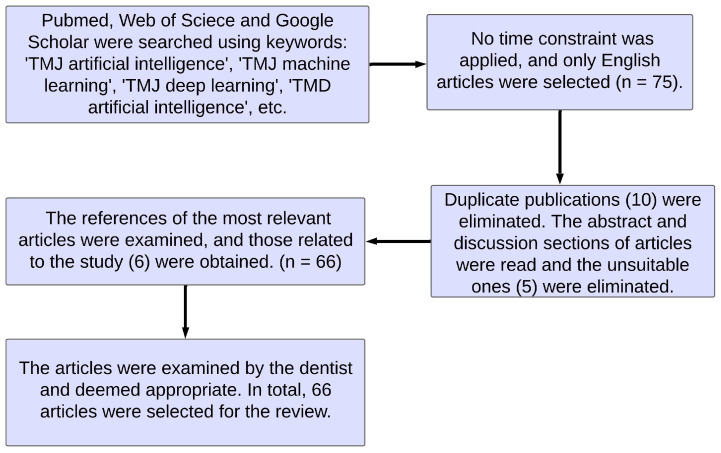
The flow chart of publication selection.

TMJ-related diseases are typically diagnosed through a patient’s clinical examination, anamnesis, and the interpretation and reporting of images by a radiologist. Nonetheless, this process can incur costs and consume a significant amount of time. To address this challenge, an automated system can be employed to assist physicians in the diagnosis process. Computer-aided approaches, especially those based on artificial intelligence, machine learning, and deep learning algorithms, have shown great promise in effectively tackling such diagnostic problems. In this study, we examined AI-based and computer-aided studies in dentistry, with a particular focus on the temporomandibular joint and temporomandibular joint-related diseases. The relevant literature searching was carried out on Web of Science, Google Scholar, and PubMed. Only studies in the English language were considered, and no date restrictions were applied. To ensure relevance and appropriateness, we checked the reference lists of the most relevant papers and selected appropriate publications. We carefully read the abstracts and discussion sections of the collected articles as they provide fundamental information about each article’s subject. Additionally, we had a dentist who is an expert in the field examine the publications obtained from the screenings. With the expert’s approval, we identified and reviewed 66 articles that were deemed suitable for this study. The process of publication selection is illustrated in Figure 2.

The remaining sections of the paper are structured as follows:

1. Fundamentals of TMJ (Section 2): Provides information about TMJ, data acquisition, data utilized in diagnosis, and automatic diagnosis.
  - Diseases and Diagnostics
  - Independent Variables and Data Capturing
2. Fundamentals of AI (Section 3): Foundational knowledge about AI, ML, and DL.
  - ML
  - DL
3. Literature on TMJ (Section 4): Computer-based studies conducted on TMJ.
  - Segmentation of TMJ
  - Audio and Image-Based Diagnostic
  - Decision Support Systems
  - Review studies on TMJ
4. Discussion (Section 5)
5. Conclusion (Section 6)

## 2. Fundamentals of TMJ

### 2.1. Diseases and Diagnostics

The temporomandibular joint is a sophisticated joint comprising different elements, such as bones, cartilage, ligaments, muscles, and a small cushioning disc between the bones. Its intricate structure and frequent use make it vulnerable to various issues and disorders. TMJ-related diseases encompass a range of medical conditions affecting the TMJ and its associated structures. These conditions can lead to jaw joint pain, dysfunction, and discomfort in the surrounding areas. Some of the typical TMJ-related diseases include TMD, TMJ OA, and JIA.

TMD is a comprehensive label that includes different conditions impacting the TMJ, such as joint inflammation, disc displacement, and degenerative alterations. These conditions can lead to symptoms such as jaw pain, restricted jaw movement, clicking or popping noises, and muscle stiffness. TMD is more common in individuals between the ages of 20 and 40, and it is also more prevalent in women than in men [11,12,13,14]. TMJ osteoarthritis is a degenerative joint condition marked by the deterioration of the cartilage within the TMJ. This ailment can result in discomfort, swelling, and limited jaw mobility. JIA is a chronic inflammatory condition that can impact the TMJ in children and adolescents. This disease may lead to joint pain, swelling, and deformities, potentially affecting jaw function.

The diagnosis of TMJ-related diseases requires a thorough evaluation by a healthcare professional, commonly a dentist, oral and maxillofacial surgeon, or orofacial pain specialist. The assessment typically encompasses medical history analysis, clinical examination, and the use of imaging techniques. The diagnosis process may include the following steps:

- Gathering Medical History: the healthcare professional will start by obtaining a comprehensive medical history from the patient, including any past jaw-related problems, dental procedures, or recent injuries that could be pertinent to the current symptoms.
- Comprehensive Clinical Assessment: a detailed examination of the jaw, face, head, and neck will be performed to evaluate the extent of movement, joint sounds, tenderness, and any indications of inflammation or swelling.
- Diagnostic Imaging: X-rays, Computed Tomography (CT) scans, or Magnetic Resonance Imaging (MRI) can be employed to capture detailed images of the TMJ and adjacent structures. These imaging modalities have the capability to detect any irregularities, such as joint degeneration, disc displacement, or fractures [15].
- Occlusion Assessment: the dentist will examine how the upper and lower teeth fit together (occlusion) to identify any malocclusion or teeth alignment issues that may contribute to TMJ problems.
- TMJ Function Tests: specific tests will be conducted to assess the functioning of the TMJ and jaw muscles. These tests aid in detecting any limitations or irregularities in jaw movement.
- Palpation: a gentle examination of the jaw area will be performed to locate tender points or areas of muscle tension.
- Pain and Symptom Evaluation: the patient will be asked about the location, intensity, and duration of pain, as well as any accompanying symptoms such as headaches or earaches.
- Exclusion of Alternative Causes: due to the potential overlap of TMJ-related symptoms with other conditions, the healthcare provider will carefully eliminate other potential causes of jaw pain or dysfunction.

Rapid completion of the diagnostic process is vital for effective treatment. Automated systems are well-suited for addressing such concerns. This study focused on investigating computer-based methods used for TMJ-related diseases, as previously mentioned. All diseases were searched using the following keywords: “artificial intelligence”, “machine learning”, and “deep learning”. For instance, the search terms used were “temporomandibular joint artificial intelligence”, “temporomandibular joint machine learning”, “temporomandibular joint deep learning”, “temporomandibular joint disorders artificial intelligence”, “temporomandibular joint disorders machine learning”, and “temporomandibular joint disorders deep learning”. Similar combinations were used for other diseases, such as TMJ OA, and JIA, during the screening process. In addition to these, TMJ segmentation, decision support systems, review articles, and publications based on sound data were included in this study.

### 2.2. Independent Variables and Data Capturing

The TMJ is a complex joint that plays a crucial role in various functions, and several independent variables can influence the development and health of the temporomandibular joint. These variables can include demographic factors (age, gender, ethnicity, and socioeconomic status), medical history, lifestyle habits (teeth grinding (bruxism), clenching, nail-biting, or chewing gum), genetic predispositions, and various other biological and environmental factors. Understanding these variables helps in developing comprehensive models for the early detection and diagnosis of TMDs. Farook et al. [16] explored the digitization of jaw movement patterns using devices and analyzed how physiological factors and device-specific variables influenced jaw movements. The outcomes of their study demonstrated that factors such as mandibular and condylar growth, kinematic irregularities in the neuromuscular system, reduced dental arches, previous orthodontic interventions, variations in habitual head posture, TMDs, fricative phonetics, partially parafunctional habits, and imbalanced occlusal contact played a role as influencing factors on jaw movement paths as variables that could cause complications. However, these factors showed limited correlation with age, gender, or dietary habits.

TMJ image capture involves obtaining detailed images of the temporomandibular joint using various advanced imaging methods. X-rays, CT scans, MRIs, and Cone Beam Computed Tomography (CBCT) are commonly employed to visualize the TMJ and its associated structures. X-rays provide two-dimensional images that allow visualization of the bones and aid in identifying joint degeneration, osteoarthritis, or structural abnormalities in the joint. CT scans offer three-dimensional images, enabling a more comprehensive assessment of the TMJ’s bony structures, cartilage, and surrounding tissues. MRI, on the other hand, provides detailed soft tissue images, making it suitable for detecting disc displacement, inflammation, and joint effusion. CBCT combines the advantages of CT and panoramic X-rays, delivering high-resolution 3D images with reduced radiation exposure compared with traditional CT scans. These advanced imaging techniques play a crucial role in diagnosing TMJ-related diseases and guiding treatment decisions, as they provide essential information about the joint’s anatomy, function, and any potential abnormalities.

In the context of MRI, images of patients’ bilateral temporomandibular joints are typically captured in both sagittal and coronal oblique planes, with the jaw in both open and closed positions. This imaging is facilitated through the use of specific sequences, such as T1-weighted, MERGE, and Proton Density (PD) sequences. Within the realm of MR images focusing on the temporomandibular joint, comprehensive insights are drawn bilaterally. Experienced radiologists assess critical aspects such as the positioning of the disc, the identification of degenerative changes in the joint’s osseous structures, and the determination of effusion presence. This assessment involves the meticulous interpretation of slices ranging from 1 to 3 mm in thickness. The application of Magnetic Resonance Imaging devices operating at strengths of 1.5 to 3 Tesla yields detailed depictions of the intricate structures comprising the TMJ. Notably, the articular disc, an essential component of the TMJ, manifests as a distinctive biconcave image with a discernible low signal intensity in MRG. The mandibular condylar bone marrow showcases its characteristics in various sequences. Specifically, it produces a uniformly high signal intensity image in both T1-weighted and PD-weighted sequences while presenting a moderately graded signal intensity image in T2-weighted sequences. Conversely, bone marrow edema is visually indicated through low signal intensity in T1-weighted or PD-weighted sequences, and a contrastingly high signal intensity in T2-weighted sequences. In the domain of TMJ-related conditions, the presence of sclerosis and fibrosis is highlighted through the projection of low signal intensity images in T1-weighted, PD-weighted, and T2-weighted sequences. In contrast, osteonecrosis showcases a heterogeneous signal image, distinguishing itself from the other conditions under investigation [17,18,19].

In the realm of tomographic imaging, for a comprehensive radiographic assessment of the TMJ, it is imperative to execute two distinct scans, specifically in the closed and open mouth positions. The closed-mouth scan serves the purpose of establishing the functional position of the condyle within the mandibular fossa, a position defined by dental criteria. Depending on the clinician’s preference, the initial imaging should exhibit a medium level of resolution (with a nominal voxel size of 0.3 mm or smaller), while ensuring that the teeth are aligned in either maximum intercuspidation or centric relation. The field of view (FOV) should stretch roughly 1 cm above the glenoid fossa’s foundation (approximately at mid-orbital level), including the mandibular dentition, for the purpose of validating the occlusal alignment of the teeth. When there are concerns regarding alterations on the joint surface, it is advisable to enhance the voxel resolution (using a nominal voxel size of 0.25–0.125). To counteract the potential loss of cortical definition due to motion artifacts, it is crucial to maintain the stability of the patient’s head during the scanning process. The open mouth scan, on the other hand, serves to ascertain the extent of motion of the condylar head during mouth opening. For this purpose, a low-resolution scan with a reduced dose should be performed, employing a narrow FOV that encompasses the glenoid fossa base and the neck of the moving mandibular condyle. Ensuring the stability of the patient’s jaw is paramount during this open-scan phase. During the image reformatting process, adopting a standardized approach is indispensable to reconstructing and exhibiting the anatomical alterations in the shape and size of the TMJ’s bone components using sectional images. This method effectively minimizes discrepancies arising from parallax on the image screen and simplifies the comparison process across diverse patients or even across varying time intervals within the same patient. Protocols devised for the reformatting of TMJ images should guarantee a comprehensive assessment of articulation in standardized jaw positions (such as closed or open mouth), effectively preventing any instances where vital diagnostic information could be inadvertently disregarded. Opting for a TMJ protocol for radiological examination does not preclude the possibility of generating additional images, such as volumetric or shaded surface displays and Maximum Intensity Projections (MIPs). The primary objective of this protocol is to establish the minimum approach essential for a comprehensive interpretation. The foundational basis for reconstructing TMJ images is the creation of Multiplanar Reformat (MPR) images, oriented obliquely sagittal (or parasagittal) to the line perpendicular to the axis or plane positioned between the medial and lateral poles of the condyle, as based on the axial plane [18,20].

In ref. [21], Ahmad et al. presented a concise summary of TMJ-related disorders and the most suitable imaging methods. For more detailed information, this study can be examined.

The data collected for automated diagnosis of TMDs plays a crucial role in ensuring the accuracy and reliability of the diagnostic process. The type and quality of data utilized for training and testing machine learning or deep learning algorithms significantly influence the system’s performance in identifying and categorizing TMD-related conditions. Automated TMD diagnostics employ diverse data types, including clinical examination results, patient medical history, imaging data (e.g., X-rays, CT scans, MRI), and patient-reported symptoms. Each data type contributes unique information about the TMJ and its associated structures, allowing the algorithm to detect specific abnormalities and patterns. The quality of the data, encompassing accuracy, completeness, and relevance, is pivotal in building robust diagnostic models. The use of clean and reliable data minimizes the risk of false positives or false negatives and enhances the overall system performance. The dataset’s size utilized for training the algorithm is of utmost importance. Larger datasets often lead to more accurate and generalizable models, while a limited dataset may result in overfitting, where the algorithm performs well on the training data but fails to generalize to new, unseen data. Ensuring diversity in the dataset, encompassing a wide range of TMD-related cases, is crucial for enabling the algorithm to handle various conditions and manifestations. A diverse dataset prevents bias towards specific types of TMDs and improves the system’s ability to recognize fewer common disorders. Currently, researchers are actively exploring the potential of machine learning and deep learning algorithms to automate the diagnostic process for TMDs. Studies have extensively investigated various data types, such as radiographic images, clinical findings, and patient-reported symptoms, to develop accurate and effective diagnostic models.

## 3. Fundamentals of AI

AI is a branch of computer science focused on the creation and development of intelligent systems that can execute tasks commonly linked with human intelligence. The aim of AI is to develop systems that can learn, reason, and adapt to new situations, similar to how humans do. Through training on vast amounts of data, AI systems can recognize patterns, make predictions, and identify relationships in the data.

### 3.1. ML

ML is a branch of AI that allows machines to learn from data and enhance their performance without explicit programming. Its main objective is to create systems that can automatically identify patterns, make predictions, and make decisions based on data.

Machine learning consists of three main categories: “supervised learning”, “unsupervised learning”, and “reinforcement learning”. The model undergoes training using annotated or labeled data, where each input is paired with its corresponding output. In contrast, unsupervised learning operates with unlabeled data and seeks to uncover patterns and relationships within the data. Conversely, reinforcement learning entails instructing an agent to interact with its environment and acquire knowledge through feedback in the form of rewards or penalties.

Traditional ML algorithms, also known as classical machine learning techniques predating the emergence of deep learning, rely on statistical methods and do not utilize deep neural networks. Some examples of these classical ML approaches are: Linear Regression, Logistic Regression (LR), Support Vector Machines (SVM), Decision Tree (DT) etc. Although traditional ML algorithms have yielded successful results, they have not exhibited the desired performance when dealing with image and audio data. The emergence of DL algorithms garnered attention following Krizhevsky and colleagues’ triumph in the 2012 ILSVRC. Subsequently, researchers have frequently employed DL approaches in their studies.

### 3.2. DL

DL is a subfield of ML, a branch of AI that focuses on training algorithms to learn patterns and representations from data. The ability to automatically learn complex features from raw data has made it a powerful and popular tool. In DL, algorithms known as Artificial Neural Networks (ANNs) are used to mimic the neural connections in the brain. These nets comprise layers of nodes (interconnected), also known as neurons, which process data and transmit it to the subsequent layer. The term “deep” refers to the existence of multiple layers in these networks, allowing them to learn increasingly abstract and complex representations of the input data as they go deeper. Deep learning has demonstrated impressive achievements across diverse applications, encompassing tasks such as image processing, speech recognition, as well as Natural Language Processing (NLP). Its ability to handle complex patterns and large-scale data has contributed to its widespread adoption in numerous domains.

## 4. Literature on TMJ

### 4.1. Segmentation of TMJ

Image segmentation is a vital process in computer vision where an image is divided into multiple segments or regions, each with similar visual characteristics. Its main purpose is to extract meaningful objects or areas of interest from the original image. Accurate segmentation empowers machines to comprehend visual data and enables the development of advanced and intelligent systems. DL techniques, specifically Convolutional Neural Networks (CNNs), have significantly improved image segmentation by automatically learning relevant features from data and handling complex visual patterns. This technique holds great importance in various computer vision applications such as object detection, image recognition, medical imaging, and autonomous driving. In summary, image segmentation plays a pivotal role in computer vision and remains a critical method for analyzing images in real-world scenarios.

Automated TMJ segmentation involves employing computer algorithms and artificial intelligence methods to automatically detect and outline the boundaries of the temporomandibular joint in medical images. This process is essential in medical imaging analysis, as it assists healthcare professionals in diagnosing and treating TMJ-related diseases and disorders more effectively. Therefore, in this study, we have also examined the segmentation studies conducted on TMJ.

Ref. [22] presented an automatic system for detecting and segmenting articular discs on magnetic resonance images, with the purpose of supporting the diagnosis of temporomandibular disorder. The proposed technique employed DL-based semantic segmentation and utilized 217 resonance images from patients exhibiting either normal or displaced articular discs. Three semantic segmentation approaches based on deep learning were assessed: 3DiscNet, U-Net, and SegNet-Basic. Moreover, two specialist orthodontists with 12 and 6 years of experience, an expert oral, and a 25-year-experienced maxillofacial radiologist independently and manually segmented TMJs from MR images. Among these algorithms, SegNet-Basic and 3DiscNet exhibited comparable results in the sensitivity, dice coefficient, and Positive Predictive Value (PPV) metrics.

**Figure 3:**
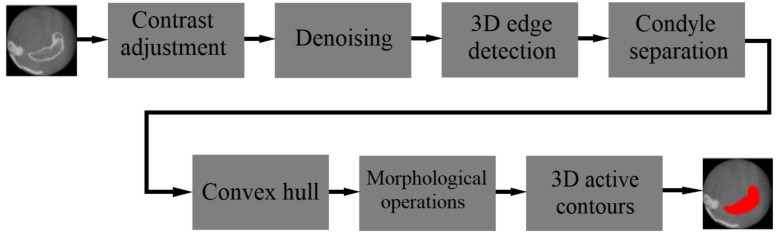
The processing workflow of TMJSeg for segmenting small field of view scans [23].

An open-source web-based software called “The Data Storage for Computation and Integration (DSCI)” was introduced in ref. [23]. DSCI introduced novel management advancements for securely storing data, deploying algorithms, and executing tasks in a web-based environment. Its design enabled the incorporation of plugins to facilitate functions such as uploading, browsing, sharing, and task execution within remote computing grids. The software offered an automatic image processing tool for TMJ segmentation using high-resolution volumetric images, automated segmentation of the mandible from CBCT images using the U-Net architecture, and automatic segmentation of digital tooth models using a model known as RUNET. Digital dental models were obtained through intraoral scanners. The model used was a modified U-Net with residual links similar to ResNet. Figure 3 [23] depicts the steps involved in segmenting small field of view scans.

Precise segmentation of the mandibular condyles and glenoid fossae is essential for quantitative analysis of TMJ volume and shape from CBCT. In ref. [24], a DL-based automated segmentation tool was presented for the exact 3D reconstruction of TMJ. They composed a 3D U-net-based three-step approach to segment the condyles and glenoid fossae. They trained and validated this method on 154 manually segmented CBCT images. Another study focusing on TMJ reconstruction is presented in ref. [25]. Additionally, for other research related to segmentation, references can be found in ref. [26,27,28,29,30,31,32,33].

### 4.2. Audio and Image-Based Diagnostic

#### 4.2.1. Juvenile Idiopathic Arthritis

JIA is a persistent form of arthritis that begins before the age of 16 and lasts longer than 6 weeks. The exact cause of the disease is not known. In dentistry, it is considered a chronic inflammatory disease that can affect the TMJ and lead to facial growth disorders, pain, and/or deterioration in jaw functions [34].

Diagnosing JIA involves a thorough assessment of the patient’s medical history, a physical examination, and laboratory tests to eliminate other potential conditions and confirm the presence of joint inflammation. Early detection and suitable treatment are crucial in preventing joint damage and enhancing long-term outcomes.

Even though MRI is widely regarded as the definitive method for diagnosing this condition, electromyography (EMG) recordings offer an alternative method for this procedure [35]. EMG provides the advantage of enabling early and immediate diagnosis. Perpetuini et al. conducted research to investigate the effectiveness of a multivariate data-driven method based on the general linear model. They examined the feasibility of this method in predicting the EMG ratio from fIRI features, where the EMG ratio (sEMG−M/T) represents the ratio between masseter and temporalis muscles. Additionally, they used the model output to distinguish between sick and healthy controls.

Among the studies conducted on JIA, refs. [36,37] are also included.

#### 4.2.2. TMJ Osteoarthritis

Temporomandibular joint osteoarthritis, a subtype of TMD, can cause various complaints, including pain, chewing dysfunction, crepitation, and dentofacial deformity [38]. It affects 5% to 12% of the population. Aging influences the increase in chronic disability in TMJ osteoarthritis, making it crucial to diagnose the condition before morphological degeneration occurs.

**Figure 4:**
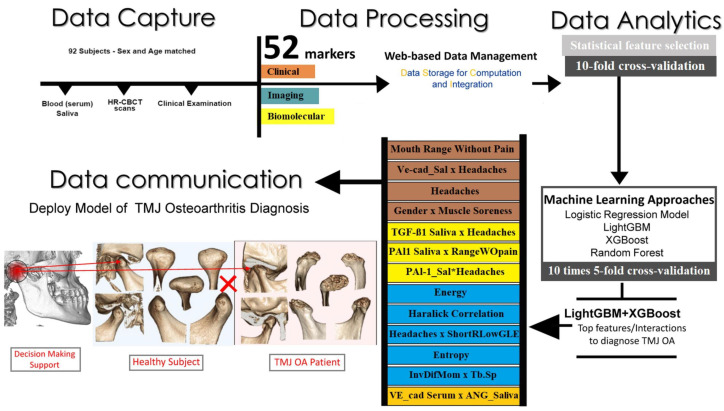
The spectrum of Data Science to advance TMJ OA diagnosis includes Data capture and acquisition, Data processing with a web-based data management, Data Analytics involving in-depth statistical analysis, machine learning approaches, and Data communication to help the decision-making support in TMJ OA diagnosis [39].

Bianchi et al. introduced a novel visualization approach in their study [39], aiming to thoroughly investigate interactions between disease and health-related biomarkers. Their data-driven approach integrated knowledge models, aiming to gain new perspectives on the intricate origins of TMJ OA. The study involved data management, clinical and biomolecular data acquisition, standardized imaging, pairing OA patients and individuals without the condition (healthy controls) based on standardized demographics, and cross-checking patient data collected from various data repositories. They evaluated 52 variables using machine learning methods to determine the condition of TMJ OA. The most relevant integrative feature pools were identified by leveraging machine learning algorithms. Standardized patient characteristics from various resources were combined using statistical machine learning algorithms, which they believed would enable an accurate diagnosis of TMJ OA condition. Figure 4 illustrates the flow chart of the method used by [39].

In the literature, various studies on TMJ OA utilizing AI approaches can be found, as referenced in the following studies: [40,41,42,43,44,45,46,47,48].

#### 4.2.3. TMD

TMD, in general terms, refers to the inconsistent movement of the disc on the articular surface of the jaw. It can be caused by various factors and may present with different symptoms, such as severe headaches, difficulty opening the jaw, and tinnitus. Patients with different articular disc displacements and deformations constitute the most significant sub-group of joint abnormalities in people with TMD.

In this section, we review studies that utilize artificial intelligence approaches in the diagnosis of TMD. Unlike other sections, we present all of the studies related to TMD since our research in this area began. Therefore, we will provide an individual explanation for each of these studies.

**Figure 5:**
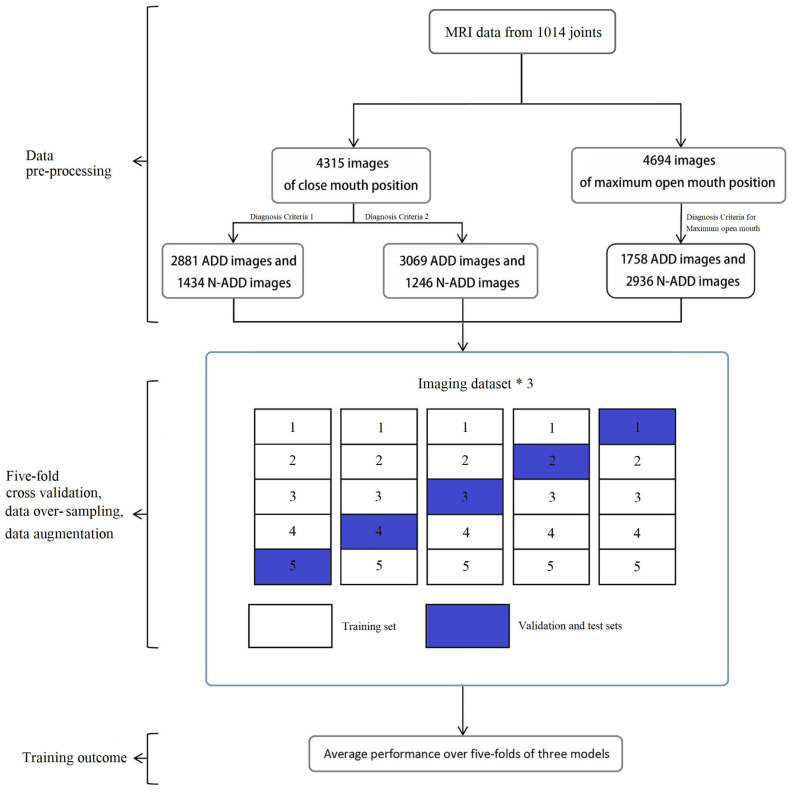
The general stages of the study [49].

The first reviewed study is [49]. autonomously identify Anterior Disc Displacement (ADD) in TMJ MR images. The main goal of this system was to mitigate the potential for severe complications following treatment. The study utilized 9009 sagittal TMJ MRIs, and accuracy and Area Under the Curve (AUC) were utilized as comparison metrics. The deep learning architecture employed was ResNet, and techniques such as 5-fold cross-validation, oversampling, and data augmentation were implemented. An overview of the study can be seen in Figure 5.

A study was conducted by Orhan et al. in ref. [50], where they investigated the effectiveness of a proposed machine learning model in classifying TMJ pathologies on MR images. The study utilized 214 TMJs from 107 patients exhibiting TMJ signs and symptoms. They employed a radiomic platform to extract imaging features related to temporomandibular joint pathologies, condylar bone changes, and disc displacements. Subsequently, feature selection, classification, and prediction were carried out by applying various ML algorithms, including Logistic Regression (LR), to the radiomic features. A total of six classifiers were employed to predict temporomandibular joint pathologies. These classifiers included DT, k-Nearest Neighbors (k-NN), SVM, logistic regression, Random Forest (RF), and XGBoost. To evaluate the efficiency of the approaches, sensitivity, specificity, and Receiver Operating Characteristic (ROC) curve metrics were utilized. Based on the experimental results, it was determined that the k-nearest neighbors and random forest classifiers proved to be the most suitable ML models for predicting temporomandibular joint pathologies.

Upon reviewing the study by Diniz et al. [51], their primary objective was to determine the efficacy of three machine learning approaches, namely SVM, k-NN, and Multi-Layer Perceptron (MLP), in feature extraction for the detection of TMD on Infrared Thermography (IT) images. For this study, a group of 78 patients was chosen according to the Fonseca questionnaire and RDC/TMD criteria. This group consisted of 37 control patients and 41 individuals who were diagnosed with TMD. In the process, the IT lateral projections of each patient were obtained, and subsequently, the masseter and temporal muscles were specifically chosen for the feature extraction procedure.

Another study examined in this review is by Kim et al. [52], which focused on the topic of temporomandibular joint disc perforation. In this research, a deep learning-based algorithm was developed to estimate the presence of TMJ disc perforation based on MRI findings. The obtained results were then compared with findings from previous studies, and the performance of the algorithm was evaluated. A total of 299 temporomandibular joints from 289 patients who were confirmed to have disc perforation during surgery were categorized into two groups based on the presence or absence of disc perforation. Expert observers interpreted temporomandibular joint magnetic resonance images to extract relevant features for the algorithm. The performances of different approaches were assessed using the ROC AUC. The MLP achieved the highest performance with an AUC of 0.940, followed by the random forest with an AUC of 0.918. In comparison, using disc shape alone resulted in a lower AUC of 0.791.

In the study conducted by Lee et al. [53], the primary objective was to employ artificial intelligence techniques to ascertain whether biological and psychosocial factors play a significant role in the development of TMDs. The study investigated factors such as stress, socio-economic status, and working conditions as potential determinants of TMDs. The dataset utilized in this research encompassed information from 4744 participants, including details on their TMD status, demographic factors, socio-economic status, working conditions, and determinants of health. To identify the factors associated with temporomandibular disorders, the researchers employed six AI methods, namely random forest, logistic regression, decision trees, naïve Bayes, SVMs, and ANN. Subsequently, the accuracy of these models was compared with evaluate their predictive performance.

Temporomandibular joint sounds, a common disorder associated with the temporomandibular joint, have drawn significant attention. In the article by Tacskiran et al. [54], a novel decision support system was proposed for diagnosing TMDs. This system relied on the integration of DL and ANN technologies. An innovative, non-invasive device was developed to record temporomandibular joint sounds, complemented by a user-friendly interface for ease of use. The dataset used in this study consisted of left and right TMJ sound recordings, ambient noise, clinical symptoms, physician’s notes regarding the patient, as well as diagnosis and treatment information. To effectively classify these measurements and determine the patient’s condition, a comprehensive approach employing signal processing, ANN, and DL algorithms was formulated. The classification success of algorithms based on frequency, statistical methods, and DL was compared. Remarkably, the DL algorithm consistently achieved success rates above 94.5%, outperforming the other two approaches. Overall, the proposed decision support system demonstrated promising capabilities in efficiently diagnosing TMDs based on temporomandibular joint sounds, showcasing the superior performance of the DL algorithm in this context.

In the study conducted by Sharma et al. [55], a neural network model was proposed to aid in understanding whether patients were experiencing temporomandibular joint disorders based on their risk factors and symptoms.

Ebadian et al. [56] investigated 200 patients aged between 20 and 50 years, with temporomandibular disorders. They explored the association of occlusal factors and parafunctional habits with TMD by utilizing Chi-square tests and an independent sample *t*-test. Additionally, they conducted binomial logistic regression analysis, taking confounding variables into consideration. Ref. [57] is another illustrative study that utilized logistic regression analysis to explore potential risk factors associated with temporomandibular joint disorders.

Another method frequently employed in studies related to TMD is Bayes’ theorem. In a study by Iwasaki [58], a Bayesian Belief Network (BBN) was applied to analyze MR images to ascertain the advancement of TMDs. The primary focus of the study was to investigate the interrelationships and impact of individual findings on one another. The dataset consisted of 295 1.5-T MRIs, representing 590 sides of the temporomandibular joint. The accuracy of the bayesian belief network was evaluated by comparing it with 11 algorithms (“necessary path condition, maximum log likelihood, path condition, Chow-Liu tree, Rebane-Pearl poly tree, greedy search-and-score with Bayesian information criterion, tree augmented naive Bayes model, K2, and C4.5, minimum description length, Akaike information criterion”), a multiple regression analysis, and an ANN using resubstitution validation and 10-fold cross-validation. Additionally, a different study utilizing the Bayes method in the context of TMD can be referenced, as cited in Ashraf et al. (2022) [59].

Lee et al. [60] conducted a study focused on the automatic detection of ADD in patients with TMD using deep learning. They acquired sagittal MRI images of 2520 TMJs and implemented data augmentation techniques to mitigate the risk of overfitting. ADAM was employed as the optimizer during the training process.

In a study by Jung et al. [61], the aim was to determine which findings on MR images of patients with TMJ Internal Derangement (ID) could serve as reliable indicators of limitations in mouth-opening and pain. Multiple logistic regression analyses were performed on the data from a total of 48 patients (96 TMJs), comprising 39 women and 9 men.

The stomatognathic system, which encompasses the digestive, sensory, and respiratory tracts, plays a significant role in the human body [10]. In the study conducted by [10], the objective was to assess the effectiveness of TMJ rehabilitation in patients. Vibration analysis, sEMG recordings of the masseter muscles, and hypertension of the masticatory muscles were employed in the evaluation process. The participants whose data were utilized in the study suffered from locking of the TMJ articular disc. Additionally, the study presented the initial findings of the k-NN method for TMD diagnosis. The research involved a group of 15 patients, comprising 10 females and 5 male individuals.

Ref. [62] employed the Self-Organizing Map (SOM) to classify patients based on their temporomandibular joint muscle activation. The application of the SOM technique was employed on a patient cohort. Together with a cross-correlation strategy, it was utilized to predict the processed surface electromyography signal acquired during TMJ muscle testing.

Another noteworthy investigation is presented in ref. [63]. The study focused on conditions that simulate TMD by exhibiting TMD-like symptoms due to non-TMD-related issues. These non-TMD symptoms arise from various pathologies and developmental or genetic abnormalities. Utilizing text mining methods including NLP and recursive segmentation, ref. [63] detected evidence-based clinical indicators that distinguish these imitative TMD conditions from genuine TMD. They examined the medical histories of 29 patients diagnosed with TMD-mimicking symptoms and contrasted them with the records of 290 patients diagnosed with authentic TMD. The study involved comparing the frequency of word usage by pre-processing the main complaints and medical histories through natural language processing. Additionally, recursive partitioning was utilized to determine the optimal mouth opening size to distinguish between fake TMD and true TMD cases.

Nocera et al. [64] conducted an implementation focused on the automatic diagnosis of patients presenting at an OroFacial Pain (OFP) clinic with various pain, headache, and TMD symptoms, employing several ML approaches. In collaboration with an expert in the field, they compiled a dataset of 451 cases by examining available electronic patient notes. The ML algorithms utilized for analysis included random forest, SVM, logistic regression, and k-NN classifiers.

Jeon et al. [65] introduced an automatic motion tracking approach for analyzing mouth opening and closing videos, investigating the relationship between the obtained results from this system and the disc position in MRI. The study used mouth opening and closing videos of 91 patients who had undergone MRI scans, captured with a digital camera. The system they devised consisted of two main phases: automatic bookmark detection of upper and lower lips from videos and graphical representations of detected locations with automatically calculated monitoring results (height and width). Disc position groups determined from MRI data were used to evaluate all monitored results. Based on the results obtained, the system was deemed reliable.

In the work of Kreiner and Viloria [66], they designed a MLP comprising a single input layer, five hidden layers, and an output layer that was trained using a backpropagation algorithm. The results for various orofacial pain categories and TMD clinical cases were compared with the diagnoses made by 12 general dental clinicians.

Furthermore, the papers [67,68] were reviewed. In ref. [67], Kao et al. conducted a study where they applied various architectures, including Inception, ResNetV2, InceptionV3, DenseNet169, and VGG16 to a dataset comprising 300 images. This dataset encompassed 52 patients with temporomandibular disorder and 32 healthy individuals. Their approach involved initially identifying the articular space between the temporal bone and the mandibular condyle through the use of the U-Net model, which was applied to 100 sagittal MRIs of the TMJ. Following this, they engaged in classification tasks employing the previously mentioned architectures. InceptionV3 achieved sensitivity, positive predictive value, accuracy rate, and F1 score metrics values of 1.0, 0.81, 0.85, and 0.9, respectively, while DenseNet169 produced values of 0.92, 0.86, 0.85, and 0.89.

Lastly, in ref. [68], the authors employed the algorithms InceptionV3, ResNet-101, Xception, MobileNetV2, ConvNeXt, DenseNet-121, and Vision Transformer (ViT) for TMD diagnosis. The dataset contained 2576 diagnostic MR images from 200 patients, categorized into four groups: “at closed mouth, articular disc position normal\in front”, “at open mouth, articular disc position normal\in front”, “joint cavity effusion”, and “mandibular condyle degeneration”. Notwithstanding the restricted availability of data, the investigation deduced that DL-based methodologies exhibited efficacy.

#### 4.2.4. Studies Using Audio Data

The investigation of TMJ sounds can provide insights into potential relationships between dental malocclusions and various sound types [69]. In their research, Akan et al. [69] recorded temporomandibular joint vibrations using accelerometers during jaw opening and closing cycles in patients presenting with lateral crossbite and Class II Division 1 malocclusion. Subsequently, the recorded signals were subjected to discrete evolutionary transformation, and time-frequency moments of the signals were derived from the evolutionary spectrum. These calculated joint time-frequency moments were utilized as features in a neural network to facilitate the classification of TMJ vibrations.

Furthermore, a separate investigation of temporomandibular joint sounds is evident in the work of Djurdjanovic et al. [70]. The assessment of sounds such as clicking and/or crepitation in the TMJ during its functional motions carries diagnostic significance. Their objective was to identify the best signal representation and pattern recognition technique for categorizing TMJ sounds. In particular, they examined and contrasted the effectiveness of time-shift invariance with and without scale invariance.

Other studies focusing on joint sound can be found in the references cited as [71,72,73].

### 4.3. Decision Support Systems

A decision support system is a computer-based information system that aids in selecting among various options, expediting the problem-solving and decision-making processes.

Due to the rapid progress of technology, there has been a substantial rise in the quantity of accumulated data. Data originating from diverse sources, such as medical devices, health records, clinical examinations, imaging, and experimental and biological data, generates a vast amount of information [74]. Effective management and analysis of this data allow for the extraction of meaningful insights that contribute to improving population health and well-being. In the study by Al et al. [74], data science approaches applied in clinical decision support systems for orthodontics were investigated. The authors also introduced a web-based data management system tailored for TMJ and dental clinical decision support systems.

The integration of developing technologies has also found application in various areas of dentistry [75]. These applications, embracing digitalization in dentistry, significantly contribute to treatment and diagnosis. Given that the diagnosis and treatment planning largely depend on the expertise of specialists, pattern recognition methods hold great promise for both physicians and patients. Machoy et al. [75] conducted an analysis of artificial intelligence applications such as Genetic Algorithms (GAs) and Clinical Decision Support Systems (CDSS) in both research and clinical dentistry.

Aiming to establish a prediction model through classification tree statistical analysis for determining the occurrence of TMD, Waked et al. [76] categorized the sample into high- and low-risk groups for disease progression. The dataset encompassed 776 individuals seeking medical or dental services from the “Family Health Units in Recife, PE, Brazil”. The samples underwent anamnesis utilizing the Research Diagnostic Criteria device for TMDs. The data underwent analysis using the software “Statistical Package for the Social Sciences 20.0”. Bivariate analysis was conducted using the Pearson Chi-square test, while multivariate analysis was performed through the utilization of the classification tree method. The occurrence of TMD was determined to be influenced by factors such as age, orofacial pain, and depression.

In addition to the aforementioned studies, other research works focusing on clinical decision support systems in dentistry include those by Mago et al. [77], Polavskova et al. [78], and Mendonca et al. [79].

### 4.4. Review Studies on TMJ

In this article, we have also explored various review studies, the first of which was conducted by Farook et al. [80]. In their work, Farook et al. investigated the clinical impact, success, limitations, and comparative results of machine learning applications in several areas of dentistry. These areas include periodontal diseases, dental diseases, cysts and tumors, trauma and neuralgias, glandular disorders, and bone and TMDs associated with dental and orofacial pain. Two reviewers systematically searched the Scopus, PubMed, and Web of Science databases until 29 October 2020. They conducted a comprehensive scan and narrative synthesis of relevant articles following the PRISMA-DTA guidelines. Moreover, the articles were evaluated by comparing them to reference tests performed by clinicians using the MI-CLAIM checklist.

Another review article under examination is authored by Bianchi et al. [81]. In this study, artificial intelligence and machine learning techniques for diagnosing temporomandibular joint osteoarthritis were reviewed, with a particular focus on data science methods employed for image processing. The authors utilized a web-based system for data storage, management, and processing.

Additional examples of studies exploring artificial intelligence applications in dentistry include those by Chen et al. [82], Shan et al. [83], Almuasan et al. [84], and Grischke et al. [85]. Moreover, Corbella et al. [86] examined deep learning applications in dentistry. Furthermore, Brickley et al. [87] investigated the applications of artificial neural network-based systems in dentistry. Additionally, review articles focusing on TMJ were considered in the works by Jha et al. [88] and Farook et al. [89].

## 5. Discussion

In recent times, there has been a notable surge of interest in the application of artificial intelligence approaches, particularly with the advent of deep learning architectures, across various sectors. The medical field has witnessed the widespread adoption of deep learning models due to their ability to achieve remarkable results, thanks to their multi-layered architecture. As a result, these models have found extensive use in medical data analysis. However, upon reviewing the literature, it becomes evident that the dental sector has seen relatively fewer studies utilizing AI methodologies.

Temporomandibular joint diseases, being among the most common disorders affecting the human body, present a complex diagnostic challenge. The diagnosis of TMJ-related diseases typically relies on the observations and interpretations made by physicians, where the expertise of the practitioner significantly influences the diagnostic process. It is important to note that these diagnostic processes are time-consuming and require a considerable amount of time from the physicians. Hence, the development of an automated diagnostic system holds great importance.

The current study explores computer-based approaches, particularly artificial intelligence methods, applied to temporomandibular joint and TMJ-related diseases. To ensure comprehensiveness, this research also encompasses applications in segmentation, decision support systems, and studies involving voice data, in addition to focusing on specific diseases. To gather relevant literature, the Web of Science, PubMed database, and Google Scholar search engine were employed. Additionally, reference lists of the most pertinent studies were searched. Notably, articles published during the preparation and submission process of this study, after the initial literature search, were not included.

Given the widespread popularity of artificial intelligence methodologies in the medical field, the primary objective of this study is to contribute to the dental literature. Furthermore, by providing an overview of the majority of relevant studies, this research aims to offer valuable insights to aid in the development of future studies and streamline the literature search process.

In Table 1, the studies are presented in groups, while Table 2 provides a more detailed presentation.

Upon scrutinizing Table 1, a comprehensive compilation of 66 studies was identified, encompassing a range of topics. This includes 11 studies that delve into segmentation, 3 that focus on JIA, 10 dedicated to TMJ OA, 21 centered around TMD, 6 investigating decision support systems, along with 10 review articles and 5 examining sound analysis. Turning to Table 2, an array of methodologies was employed across these studies to tackle the research questions. However, it is conspicuous that DL-based algorithms found application in only 7 of the segmentation studies, 4 research papers concerning TMJ OA, and 5 articles pertaining to TMD. This indicates a relatively selective adoption of DL methodologies within these domains, warranting further exploration and potential integration in future investigations.

In the realm of segmentation studies, the U-Net architecture has risen to prominence due to its noteworthy efficiency and accuracy, being implemented in 6 out of 7 cases. Additionally, the SegNet architecture was employed in two studies, with an expected broadening of its utilization in forthcoming investigations. Concerning segmentation studies, it was predicted that there would be a prevalence of DL-based approaches due to the limitations of classical machine learning methods. The research landscape on juvenile idiopathic arthritis is marked by scarcity, with only a handful of studies predominantly relying on machine learning approaches. Within the domain of TMJ OA research, deep learning methodologies have gained a stronger foothold compared with traditional machine learning techniques. Conversely, in TMD investigations, traditional machine learning approaches have held a more dominant presence, whereas the utilization of deep learning approaches has been relatively less frequent. This field demonstrates an evident shift towards embracing deep learning-based methodologies over traditional machine learning models. When examining studies pertaining to voice analysis, the deployment of signal processing approaches is discernible. However, the concurrent utilization of both imagery and audio for diagnostic purposes lacks substantial exemplars within the current literature. It is worth noting that certain ML and DL approaches have not been applied at all, leaving room for exploration. For example, architectures such as Xception [90] and Inception [91] can be considered for TMJ OA and JIA.

Another notable point is that transformers and ViTs were only used in a single study. The ViT stands for an innovative deep learning architecture that translates the foundational principles of transformers into the realm of computer vision. Initially conceptualized for natural language processing tasks, transformers gained notable prominence for their transformative impact. Subsequently, this architectural paradigm was extended to diverse fields, encompassing computer vision, which led to the formulation of ViT. Transformers epitomize attention-based [92] neural network structures, meticulously devised to apprehend intricate associations among components within sequential data. The foundational transformer architecture encompasses an attention mechanism, facilitating the computation of a weighted summation of values across the sequence. This mechanism empowers the model to concentrate on pertinent facets of the input while concurrently accounting for all elements. This inherent capability endows transformers with the adeptness to capture extensive contextual dependencies within data, rendering them adept for tasks entailing sequential or contextual comprehension. In the context of computer vision, the ViT architecture orchestrates the division of images into non-overlapping patches of consistent dimensions. These patches are subsequently subjected to linear embedding and treated as sequences. Augmenting the patch embeddings with positional embeddings confers spatial awareness upon the model. The embedded patches then undergo processing within the transformer framework, featuring self-attention mechanisms that apprehend interrelations among distinct patches. The conclusive output of this model is then harnessed for a spectrum of visual tasks, encompassing image classification, object detection, and segmentation.

It is evident from the tables that the number of publications on TMJ and TMJ-related diseases is relatively limited. However, with the increasing popularity of artificial intelligence-based approaches, especially deep learning, we can anticipate a growth in this number in the coming years.

The main reason for the lower number of studies in this field compared with other areas is the data. The effectiveness of neural networks heavily relies on the availability of diverse and high-quality data. To learn and generalize effectively, neural networks typically require a substantial amount of labeled data. However, the limited availability of data for training can lead to overfitting, where the network performs poorly on unseen data. Moreover, the generalization capability of neural networks improves when the training data encompasses various scenarios and variations, making diversity in the data crucial. Without diversity, the network’s capacity to handle unseen situations may be limited. Ensuring a diverse and representative training data set is essential for achieving reliable and accurate neural network models. A major challenge in this field is the difficulty in creating a data set, as data access is restricted by data protection measures, resulting in an insufficient number of samples. To overcome data challenges in neural networks, researchers and practitioners adopt several strategies, including data augmentation, transfer learning, pre-trained models, and adversarial training. Data augmentation artificially expands the training data by applying diverse transformations to existing samples. These transformations are typically designed to be realistic and relevant to the domain of the data. Common data augmentation techniques in the context of computer vision include image rotation, flipping, scaling, translation, shearing, zooming, and color jittering. In addition to this, another data augmentation method is the generation of synthetic data with Generative Adversarial Networks (GAN). Data augmentation using GANs is an innovative and advanced approach that exploits GANs’ capabilities to produce realistic and diverse synthetic data for data augmentation. This cutting-edge technique shows great potential for improving the training of deep learning models and addressing data scarcity in various fields, including NLP, computer vision, and medical imaging. GANs are composed of two competing networks, the generator, and the discriminator, trained together to generate synthetic data. However, it is important to acknowledge that data augmentation with GANs presents some challenges. Generating high-quality synthetic data requires a well-trained GAN, and at times, GANs may generate unrealistic or noisy samples. Ensuring the reliability and diversity of the generated data is essential for the success of this approach [93]. The studies by Chlap et al. [93] and Mikolajczyk et al. [94] have extensively examined data augmentation techniques, providing comprehensive discussions on the subject. Researchers interested in acquiring insights into data augmentation methods may find these studies valuable resources for exploration and understanding. Transfer learning involves utilizing knowledge gained from one task or domain to improve performance on another task or domain that is related but different. Pre-trained models involve utilizing networks trained on extensive datasets for general tasks, such as image recognition, and fine-tuning them for specific tasks with smaller datasets. Adversarial training aids in enhancing the model’s robustness to minor perturbations and improving its overall generalization capability.

Another issue is that artificial networks are black boxes. Traditional neural networks, especially deep learning models, are often regarded as “black boxes” due to their utilization of intricate mathematical transformations and high-dimensional data, leading to challenges in comprehending their decision-making process. Despite their remarkable performance in tasks such as image recognition, NLP, and speech recognition, the growing complexity of networks poses challenges in comprehending their predictions. As the networks learn from input data and desired outputs, they adjust their internal parameters (weights and biases), resulting in highly intricate structures that are hard to interpret. This complexity hinders the identification of specific features or patterns in the data that contribute to specific predictions. The lack of interpretability poses a significant concern in critical fields such as healthcare, finance, and autonomous vehicles, where transparent and explainable decision-making processes are crucial. To address the black box problem, researchers have proposed “Explainable AI” (xAI), an area of AI research focused on enhancing the transparency and interpretability of AI systems for humans. The primary aim of xAI is to provide meaningful explanations for the decisions and predictions made by AI models, empowering users and stakeholders to better comprehend the reasoning behind the results. This becomes especially vital in domains where the consequences of AI decisions have substantial impacts on individuals and society. Some common techniques employed in xAI include feature visualization, feature attribution, rule-based models, saliency maps, and contrastive explanations. These techniques aim to shed light on the inner workings of AI models and provide insights into the factors influencing their predictions, enabling users to build trust and confidence in the AI systems’ decisions. By incorporating xAI approaches, AI systems can become more interpretable, transparent, and trustworthy, ensuring their responsible and effective application across various critical applications.

Although there are some challenges to overcome, it is anticipated that DL algorithms will play a more noteworthy role in this field. The future holds promise for the automation of inferences through the implementation of DL applications on dental images. Our current work is focused on TMD, aiming to contribute to the existing literature and support dentists in making accurate diagnoses. As this study centers on TMD, we have provided an overview of all computer-assisted or artificial intelligence-based studies related to TMD in this article. However, in future review articles, we can delve into the individual successes of each study, evaluating their effectiveness and identifying any limitations they may have. By comparing similar subjects and highlighting shortcomings, we can assess the overall progress in this field. Furthermore, AI, particularly ML and DL-based algorithms, has had a significant impact on medical applications. The rapid development of new algorithms in this domain has been remarkable. We believe that our research will serve as a starting point for future studies to create more accurate and reliable models for TMJ-based diseases. These advancements will undoubtedly improve patient care and contribute to the field of dental healthcare.

## 6. Conclusions

In recent times, rapid advancements in technology have revolutionized various fields, including the medical domain, where computer-based investigations and AI-based algorithms have gained substantial importance. The prevalence of TMJ-related diseases affecting a frequently utilized joint in the human body should not be underestimated. The present study aimed to explore computer-based and artificial intelligence-based approaches employed in TMJ and TMJ-related diseases, aiming to contribute to the existing literature and pave the way for future research endeavors. However, upon evaluation, it became evident that the number of studies conducted in this area remains insufficient. The underlying reasons for this limitation were examined, and potential solutions were elucidated. Encouragingly, considering the recent increase in published papers and the ongoing efforts to address these challenges, it is anticipated that novel studies in this domain will emerge, leading to a promising growth in their quantity and quality.

## Figures and Tables

**Table 1:** Summary of studies.

Segmentation	JIA	TMJ OA	TMD	Decision Support Systems	Review	Sound
[22]	[37]	[39]	[49]	[74]	[80]	[69]
[23]	[36]	[40]	[50]	[75]	[81]	[70]
[26]	[35]	[41]	[51]	[77]	[82]	[71]
[27]		[42]	[52]	[78]	[83]	[72]
[28]		[43]	[53]	[79]	[86]	[73]
[29]		[44]	[62]	[76]	[84]	
[24]		[45]	[54]		[85]	
[25]		[46]	[55]		[87]	
[30]		[47]	[56]		[88]	
[31]		[48]	[57]		[89]	
[32]			[58]			
			[59]			
			[60]			
			[61]			
			[10]			
			[63]			
			[64]			
			[65]			
			[66]			
			[67]			
			[68]			

**Table 2:** Summary of data sets and methods (Seg. = Segmentation).

Author	Data Set	Method	Outcome	Category
[22]	217 MRIs (106 from the 10 patients (between 19 and 39 years) and 111 from the 10 control subjects (between 18 and 41 years)).	3DiscNet, U-Net, Seg-Net Basic. No preprocessing, no augmentation. For 3DiscNet, ADAM optimizer, 1.0 × 10${}^{-3}$ learning rate. For all algorithms, 2000 epoch.	For normal, 3DiscNet had (0.76 ± 0.08) dice, (0.73 ± 0.12) sensitivity, and (0.81 ± 0.11) PPV For displacement, 3DiscNet had (0.70 ± 0.17) dice, (0.72 ± 0.19) sensitivity. SegNet-Basic had (0.76 ± 0.16) PPV	Seg.
[23]	CBCT scans with small FOV and large FOV. %20 for test, 10-fold cross validation.	U-Net, RUNET (UNET with residual connections similar to RESNET). Contrast adjustment, Canny edge detection, and contour techniques. No augmentation, 60 epochs, batch size of 8, and $10^{-5}$ learning rate.	Average 0.95 Dice coefficient	Seg.
[26]	187 3D CBCTs scans of 95 patients. 94 left condyles, 93 right condyles	Denoising, 3D active contour and morphological operations.	A Dice coefficient of 0.9461 accompanied by a standard deviation of 0.0888	Seg.
[27]	CBCT images of 20 adolescent subjects.	Marker-based watershed transform.	The Dice coefficient was 0.97 ± 0.01	Seg.
[28]	1200 MRIs (600 TMJs of 357 patients). 1000 MRIs from Hospital A (800 of them for training, 200 of them for internal validity test). 200 MRIs from Hospital B (All of them were used for external validaiton).	U-Net (from a Neural Network Console) No preprocessing, no augmentation. 300 epochs, 0.001 initial learning rate, and ADAM solver.	The evaluation of effectiveness utilizing data from Hospital B revealed reduced sensitivity compared with Hospital A, with both achieving accuracy rates exceeding 80%. Precision was diminished in utilizing Hospital A’s test data for ascertaining the anterior disc displacement position. In terms of classifying intra-articular TMD, a greater ratio of accurately categorized TMJs was observed with the utilization of images from Hospital A, surpassing those obtained from Hospital B.	Seg.
[29]	160 training MRIs of 4 persons (80 positive and 80 negative). 10-fold cross validation	19 different approaches (Viola-Jones, Auto Color Correlogram measure, CEDD, Color Layout, Edge histogram, FCTH, Fuzzy Colour Histogram, Gabor, General Color Layout, IPEG Coefficient Histogram, Simple Color Histogram, Tamura, Scalable Color, SVM comb-radial kernel, SVM comb-dot kernel, SVM comb -linear kernel, k-NN (k = 1, Canberra measure), Decision trees, SVM comb. -Gaussian kernel, SVM comb.-Neural network, SVMcomb.-Anova). No augmentation	The highest accuracy was attained by employing a Gaussian kernel-based SVM method (accuracy: 98.16 ± 2.81%).	Seg.
[30]	A total of 206 CT scans with reduced radiation dosage were employed. Of these, 123 3D images were utilized for training, 42 for validation, and 41 for the test dataset. Subsequently, the images were divided into individual 2D slices, resulting in 10,313 2D images for train, 3502 for val., and rest 3351 for the test.	U-Net+tracking-based algorithm. Normalization, horizontal flip and random rotation were applied. ADAM algorithm, 0.01 initial learning rate, discard rate is 0.5 for dropout layer, and model was obtained after 43 iteration.	DCs = 0.92 ± 0.03 (condyles) and 0.90 ± 0.04 (glenoid fossae), and MSDs = 0.20 ± 0.19 mm (condyles) and 0.19 ± 0.08 mm (glenoid fossae).	Seg.
[31]	12,800 CBCTs from 25 subjects, 18 subjects (9216 slices) for the training, 5 subjects (2560 slices) for the validation, 2 subjects (1024 slices) for the test set.	U-Net-based model. Adam optimizer with a fixed momentum (0.9, 0.999), weight decay of $10^{-4}$, a batch size of 0.1, learning rate of $10^{-4}$. 50 experiments using Monte Carlo cross validation with 30 epochs.	The IoU and Hausdorff distance accuracy were 0.870 and 0.928 for marrow bone and 0.734 and 0.247 for cortical bone, respectively.	Seg.
[32]	49,094 CBCTs of 102 patients (aged 18–90 years). 6:2:2 ratio for train:validation:test.	U-Net, SegNet. For 2D network, 600 epochs, ADAM optimizer, learning rate and decay (0.01, 0.005) for SegNet, and (0.0001, 5 × 10${}^{-4}$) and a momentum of 0.9 for U-Net. For 3D network, 100 epochs, ADAM optimizer, learning rate 5 × 10${}^{-4}$(decay factor 5), batch size of 8. No augmentation, preprocess was applied.	The accuracy achieved by the 2D U-Net utilizing neighboring images was 0.82. The 2D SegNet exhibited an accuracy of 0.96, while the 3D U-Net demonstrated the highest accuracy of 0.99.	Seg.
[24]	130 manually segmented CBCTs for train 24 for validation, and 8 CBCTs for test.	3D U-Net model. ADAM optimizer, decaying triangular cyclic learning rate, 5 × 10${}^{-3}$ the base learning rate, 2 × 10${}^{-3}$ the max learning rate, 2000 step size. Batch size of 8 for the first 3D U-Net, batch size of 4 for the other two 3D U-Nets. Batch normalization with a momentum of 0.9, a dropout rate of 0.2.	The condyles and glenoid fossa achieved IoU scores of 0.955 and 0.935, respectively.	Seg.
[25]	Patient’s frontal face photograph	AI-based method	-	Seg.
[35]	EMG data, 23 healthy participants and 9 TMD patients (18 males, age range 5–17 years, mean 12.4 years)	Multivariate data-driven approach based on General Linear Model (supervised ML). Bootstrap (1000 iterations) was applied. No augmentation, no preprocessing	AUC of 0.71	JIA
[37]	Immune signature 85 patients diagnosed with JIA and 43 controls matched for age. 10-fold cross validation	Random forest, ANN, SVM Hyperparameters for the algorithms were determined by experiment. No preprocessing, no augmentation.	A ∼90% accuracy was achieved in distinguishing patients with JIA from healthy controls.	JIA
[39]	CBCT 92 patients, 46 TMJ OA and 46 control subjects matched for age and gender 5-fold cross (10 times) validation	Logistic regression, random forest, LightGBM, XGBoost. No augmentation	The XGBoost and LightGBM ensemble model attained an accuracy of 0.823, an AUC of 0.870, and an F1-score of 0.823 for the diagnosis of TMJ OA condition.	TMJ OA
[40]	314 patients, 3514 sagital CBCTs for train 300 images in total (2 sets) for test	a Single-Shot Detector (SSD) model No augmentation, no preprocessing	For the two test sets, the mean accuracy, precision, recall, and F1 score were 0.86, 0.85, 0.84, and 0.84.	TMJ OA
[41]	1189 OPG, 2378 joints, 800 are normal, 779 are indeterminate, and 799 are OA (based on the CBCT)	Inception ResNet V2 Data augmentation was done by image rotation, image shift, brightness, and contrast, no preprocessing. 700 epochs, learnin rate of 1.0 × 10${}^{-6}$, ADAM optimizer	Accuracy: 0.78, sensitivity: 0.73, and specificity: 0.82 (When the indeterminate OA was removed).	TMJ OA
[42]	A collective sum of 858 panoramic images of the TMJ were included, comprising 395 images classified as normal and 463 images categorized as TMJ-OA. These images were obtained from a cohort of 518 individuals. Ratio of train, validation, and test was (6:2:2).	Resnet152 and EfficientNetB7 learning rate of $10^{-4}$, 1000 epochs, ADAM optimizer, and a weight decay of $10^{-4}$. Augmentation techniques: Horizontal flip, color jitter, random affine, random rotation. ROI preprocessing.	The classification accuracies of ResNet152 and EfficientNetB7 were 0.87 and 0.88, respectively.	TMJ OA
[43]	17 TMJOA patient, 17 control subjects 259 condyles were used for training, 34 for test.	a NN, No augmentation.	The accuracy of the NN’s staging of TMJOA in comparison to the repeated clinicians’ consensus was found to be 73.5% and 91.2%, respectively.	TMJ OA
[44]	293 CBCTs in total (259 training set and 34 testing set). In training, 105 control subjects and 154 patients of TMJ OA.	The shape variation analyzer (implemented as a deep neural network) utilized a NN architecture consisting of 4 hidden layers with neuron counts of (4096, 2048, 1024, 512), along with a dropout layer with a dropout probability of 0.5. The network also incorporated a softmax layer with 7 output units. The learning rate was configured as $1^{-5}$, and the training process spanned 100 epochs, employing a batch size of 32. Augmentation was applied, no preprocessing	They achieved an exact classification accuracy of 47%. However, if they allowed for an error of +/−, the accuracy increased to 91%.	TMJ OA
[45]	92 subjects, 46 TMJ OA patients, and 46 healthy controls 5 fold cross valiation	TMJOAI (a diagnostic tool) has three parts: The feature preparation, selection and model evaluation.	The optimal performance was attained by averaging the predictions of the XGBoost and LightGBM models. Furthermore, the incorporation of an additional 32 markers from the mandibular fossa of the joint resulted in an enhancement of the prediction performance of the AUC, increasing it from 0.83 to 0.88.	TMJ OA
[46]	Panoromic images, 1292 patients For condyle detection (800 train, 167 test) For condyle discrimination (2066 train, 518 test) For condyle detection (1154 train, 778 test)	R-CNN for learning, VGG16 for condyle discrimination Duplicating and rotating. No preprocessing	The R-CNN achieved an average precision of 99.4% (right side) and 100% (left side) for condyle detection at intersection over union (IoU) > 0.5. As for the TMJ OA classification algorithm using a CNN, its sensitivity, specificity, and accuracy were 0.54, 0.94, and 0.84, respectively.	TMJ OA
[47]	CBCT The training dataset included 259 condyles, with 105 condyles sourced from control subjects and 154 from TMJ OA patients. For testing purposes, 34 condyles (17 right and 17 left) from a total of 17 patients were used.	A classifier based on a deep neural network for 3D condylar morphology (referred to as SVA), along with a versatile web-based system designed for data storage, computation, and integration (referred to as DSCI). 1 hidden layer, 2001 iterations, 50 epochs	The SVA classifier achieved a close agreement of 91% with the clinician consensus.	TMJ OA
[48]	A total of 2000 sagittal sections were extracted from CBCT DICOM images of 290 patients. These sections consisted of 500 images each for the categories of healthy, erosion, osteophyte, and flattening.	YOLOv5, 500 epoch No augmentation, no preprocessing	The model’s sensitivity, precision, and F1 scores for TMJ OA classification were 1, 0.7678, and 0.8686, respectively, with an accuracy value of 0.7678. The classification model’s prediction values were 88% for healthy joints, 70% for flattened joints, 95% for joints with erosion, and 86% for joints with osteophytes. As for the YOLOv5 model for TMJ segmentation, its sensitivity, precision, and F1 score are 1, 0.9953, and 0.9976, respectively, while the AUC value is 0.9723.	TMJ OA
[10]	Vibration and sEMG signals, a measurement system was developed, and sample outcomes from the rehabilitation process of a 27-year-old female patient with temporomandibular joint articular dysfunction are showcased. 15 patients(ten women and five men) for test.	k-NN k = 1	The k-NN algorithm produced results ranging from 62.5% to 82.1%. In the opening with protruding exercise, effectiveness of 71.1% and 75.0% was achieved using vibration and EMG, respectively, while the combined classification achieved 79.1% effectiveness. After data fusion, the recognition efficiency for fast opening increased from 73.6% to 85.3%. However, the results for the remaining exercises, slow opening, and slow protruding, were not as promising.	TMD
[49]	507 patients (426 female, 81 male) 9009 MRI %80 train, %10 validation, %10 test 5-fold cross validation	ResNet34, learning rate 0.001, 20 rounds of training Horizontal flipping, 10% zooming, 10% shifting	Theopen mouth position model exhibited accuracy and AUC of 0.970 (±0.007) and 0.990 (±0.005), respectively. The closed mouth position models, diagnostic Criteria 1 demonstrated higher accuracy and AUC with values of 0.863 (±0.008) and 0.922 (±0.009), respectively, which were significantly superior to those of diagnostic Criteria 2 (0.839 ± 0.013, *p* = 0.009; AUC 0.885 ± 0.018, *p* = 0.003).	TMD
[50]	MRI, 214 TMJs from 107 patients %80 train, %20 test	Logistic regression, random forest, decision tree, k-NN, XGBoost, SVM	The k-NN and RF classifiers were identified as the most effective machine learning models for predicting TMJ pathologies.	TMD
[51]	IT, 78 patients (37 control and 41 TMD) %70 training, %30 test cross validation used	k-NN, SVM and MLP k = 5 (for k-NN), The 2. layer has 8 neurons, the 3. and 4. layers have 6 neurons, the 5. has 4 (for MLP) Hopkins’s statistic, Shapiro–Wilk, ANOVA and Tukey tests were used, the significance level was 5% (*p* < 0.05).	Statistically significant differences were observed in the accuracy, precision, and sensitivity values between the semantic and radiomic-semantic associations compared with the radiomic features (*p* = 0.008, *p* = 0.016, and *p* = 0.013, respectively).	TMD
[52]	MRI, 299 joints from 289 patients (168 non perforated, 131 perforated) %80 train, %20 validation	Random forest, MLP No augmentation, no preprocessing	The MLP demonstrated the highest performance with an AUC of 0.940, followed by random forest with an AUC of 0.918, and disc shape alone with an AUC of 0.791.	TMD
[53]	4744 participants’ 37 independent variables of TMDs (demographic factors, working conditions, socioeconomic status, and health-related determinants). %75 train, %25 test	Decision trees, logistic regression, random forest, naïve Bayes, SVM and an ANN The decision tree employed GINI, the random forest consisted of 1000 trees, radial basis function was utilized as the kernel for the SVM, and the ANN featured 2 hidden layers (10-10) with quasi-Newton (lbfgs) as the weight optimization method.	It has been observed that the factors selected by the Random Forest algorithm are similar to those chosen by the doctor.	TMD
[54]	Sound data 48 different individuals (21 healthy and 27 TMJ patients). 76 data (48 healthy, 28 TMD) For ANN, 54 training, 11 validations, 11 test For DL, the data was divided by 3 and the number was tripled.	Signal processing, ANN, DL For ANN, 10 hidden layers, sigmoid activation function For DL, each intermediate layer consists of a Convolution2DLayer, a ReLU-Layer, and a Maxpooling2DLayer. After the intermediate layers, there is a fully connected layer, a softmax layer, and a classification output layer.	The average success rate of the frequency-based feature extraction method using ANN classification is 78.6%. For the statistical-based feature extraction method, the average success rate with ANN classification is 89%. Additionally, the average success rate of the deep learning-based method is 94.5%.	TMD
[55]	The dataset comprises information from 2300 patients with TMJ syndrome, presenting symptoms such as locking of the jaw or limited movement, painful clicking, popping, or grating sounds, alterations in the fitting of upper and lower teeth, radiating pain in the face, jaw or neck, aching pain in and around the ear, jaw muscle stiffness, and underlying factors like genetic predisposition, diabetes, parathyroid issues, and vitamin D deficiency.	ANN 17 epoch, 20 neurons in hidden layer (1 hidden laye)	98.9% of the correlation coefficient in terms of regression curve for the TMJ disorder prediction	TMD
[56]	200 patient (patients between 20 and 50 years) Demographic information includes variables such as age, gender, and education level. Parafunctional aspects encompass bruxism and clenching behaviors, as well as habits such as nail biting and gum chewing. Occlusal factors involve dental relationships, lateral occlusion scheme, horizontal disparities between centric occlusion and maximal intercuspation (MI), as well as discrepancies between MI and the mandibular resting position.	The analysis of TMD was conducted using Chi-square tests and independent sample *t*-test at a significance level of α = 0.05. Additionally, binomial logistic regression analysis was carried out, taking into account potential confounding variables.	The prevalence of TMD was found to be 58.9%. Among the parafunctional and occlusal factors examined, only bruxism demonstrated a statistically significant difference between the TMD and non-TMD groups (p< 0.05). However, other parafunctional and occlusal factors did not exhibit significant influence on the occurrence of TMD.	TMD
[58]	MRI 295 cases, and 590 right and left sides of TMJs (54 male; 241 female) 10-fold cross-validation	The accuracy of the Bayesian Belief Network (BBN) was evaluated by comparing it with 11 different algorithms, (including “necessary path Rebane–Pearl poly tree, condition, Chow–Liu tree, path condition, greedy search-and-score with Bayesian information criterion, tree augmented naive Bayes model, minimum description length, maximum log likelihood, Akaike information criterion, K2, and C4.5), a multiple regression analysis and an ANN” using resubstitution validation and 10-fold cross-validation.	The BBN path condition algorithm using resubstitution validation and 10-fold cross validation was 0.99% accurate.	TMD
[59]	Survey (9922 invited participants aged 18 years or older) The main dataset utilized in the study consisted of 530 individuals (information of clinical oral examination, severe headaches). From the main dataset, they created dataset 1 (*n* = 345) and dataset 2 (*n* = 464)	Bayesian logistic regression models	The presence of migraine at follow-up was not associated with either of the baseline TMD-related pain variables (posterior effect estimates: −0.12, 95% credible interval [CI] −0.49– 0.24, and 0.11, 95% CI −0.38–0.59, for mTMD and jTMD, respectively). However, mTMD at baseline was associated with the presence of tension-type headache (TTH) at follow-up (posterior effect estimate 0.36, 95% CI 0.02–0.69), whereas jTMD at baseline was not significantly associated with TTH at follow-up (posterior effect estimate −0.32, 95% CI −0.94–0.25).	TMD
[60]	MRI images of 2520 TMJs (861 men, 399 women; average age 37.33 ± 18.83 years). 2051 for train, 468 for test, %20 of train for validation	VGG16 (fine-tuning, from scratch and freeze were evaluated separately) 3 different augmentations (randomly flip in vertical and affine transform image. Contrast changes: randomly changed contrast values and applied histogram equalization. Brightness changes: randomly changed brightness of image). Learning rate of 1 × 10${}^{-4}$ for the fine-tuning and from-scratch (their epochs were 15 and 30 respectively), the freeze model utilized 5 × 10${}^{-4}$ with 150 epochs. All 3 had the ADAM optimizer.	The fine-tuning model achieved a prediction performance with an AUC of 0.8775 and an accuracy of 0.83%. The AU C values for the from-scratch and freeze models were 0.8269 and 0.5858, respectively.	TMD
[61]	MRI, clinical examinations for pain and mouth opening limitation. 96 TMJS (48 patients, 39 female and 9 male)	Multiple logistic regression	The data analysis revealed statistically significant correlations between pain and the degree of disc displacement on MR imaging (p< 0.05). The likelihood of experiencing pain in moderate to significant cases was 9.69 times higher compared to normal cases.	TMD
[62]	sEMG signals from the TMJ muscles during jaw movements 42 volunteers participated	Self-Organizing Map (SOM) combined with cross-correlation analysis	Intra cross-correlation coefficient (CC) provided information about muscle activity similarity within and between patients, indicating TMJ function stability. SOM analysis helps interpret muscle activation and assess TMJ health, while cross-correlation identifies similarities in sEMG data between patients, potentially aiding clinical TMD diagnosis and treatment assessment	TMD
[64]	451 cases (320 females and 131 males) from patient notes between 8 and 93 years old, average age is 43.4 years. 141 variables (3 continuous, 138 dichotomous: age, pain severity and max mouth opening in millimeters) 5-fold cross validation	High Frequency Value (HFV) multilabel, random forest multilabel, random forest, Logit, SVM, and k-NN HFV method used THF = 0:67, random forest had 100 trees, SVM used a slack variable cost C = 1 and Logit used a SAG solver.	The accuracy varies between 75.60% and 96.8%, recall varies between 46.27% and 98.21%, precision varies between 52.40% and 97.38% and the F-1 score ranges from 0.56 to 0.96.	TMD
[63]	Medical records of 290 patients (61 male and 229 female; average age, 31.2 ± 15.8) with genuine TMD and 29 patients (14 male and 15 female; average age, 39.5 ± 23.2) with TMD-mimicking conditions. 10-fold cross validation	natural language processing and recursive partitioning.	The model exhibited a predictive performance of 96.6%, achieving a sensitivity of 69.0% and a specificity of 99.3% in anticipating TMD-mimicking conditions.	TMD
[67]	MRI of 52 patients with TMJ Disc displacement (TMJDD) and 32 healthy controls. 300 images (105 normal, and 195 patient) 5-fold cross validation 100 MRI used for U-Net	U-Net for joint cavity detection and InceptionResNetV2, InceptionV3, DenseNet169, and VGG16 for classification.	Among the models evaluated, InceptionV3 and DenseNet169 demonstrated the best performance. In the case of InceptionV3, the recall, precision, accuracy, and F1 score were 1, 0.81, 0.85, and 0.9, respectively. Similarly, for DenseNet169, the corresponding values were 0.92, 0.86, 0.85, and 0.89.	TMD
[65]	Mouth opening and closing videos of 91 patients (17 males and 74 females; mean age, 38.42 years old) The graph paths were: straight, sideways-skewed, and limited-straight line graphs	The automated model encompasses the following components: (I) the creation of an automated system for detecting landmarks on both the upper and lower lips within the video footage; and (II) the generation of a visual representation of the tracing graph and the subsequent automatic computation of both the graph’s height (indicating mouth opening length) and width (representing sideways values).	Subjects with a normal disc position exhibited mainly straight line graphs in 85.72% of cases. The presence of sideways skewed or limited -straight line graphs was identified in 85.0% of cases within the anterior disc displacement with reduction group and in 89.47% of cases within the anterior disc displacement without reduction group. This observation exhibited a statistically significant correlation of (χ2 = 38.113, p< 0.001) between the two groups.	TMD
[66]	Clinical cases	MLP consisted of an input layer with 18 neurons, followed by 5 hidden layers, and concluded with 1 output layer. Backpropagation training	The accuracy of the MLP outperformed that of the general dental clinicians, with a statistically significant difference (*p* = 0.0072).	TMD
[68]	2576 MR images of 200 patients (4 classes) %80 for train, %20 for test and %20 of training for validation Contrast, flip and rotation augmentation methods were applied.	Performance of a CNN, fine-tuned Xception, ResNet-101, MobileNetV2, InceptionV3, DenseNet-121 and ConvNeXt, and transformers were evaluated. 100 epochs, binary cross-entropy loss function, ADAM optimizer and 1 × 10${}^{-6}$ learning rate.	It was determined that MobileNetV2 exhibited the best performance for the “closed mouth disc position” group, while Xception performed the best for the “open mouth disc position” group. In terms of “joint cavity effusion,” ResNet-101 showed superior results, and for the “mandibular condyle degeneration” group, MobileNetV2 demonstrated the highest performance.	TMD
[57]	Gender, economic class, age and marital status. 1000 patients (aged 15 to 70, 57% were over 30 years of age and 83% were women. 53% were not married) were divided into 3 class which were Class A (high social class), Classes B/C (middle class) and Classes D/E (very poor social class).	Pearson’s chi-square test for proportions, fisher’s exact test, nonparametric mann-whitney test and binary logistic regression analysis	No participants belonged to Class A, 72% belonged to Classes B/C and 28% belonged to Classes D/E	TMD
[69]	TMJ sound data 11 orthodontic patients (22 joints) aged between 9 and 13 years old with lateral cross-bite, as well as 21 orthodontic patients (42 joints) within the age range of 9 to 13 years old with Class II Division I type malocclusions.	Discrete evolutionary transform, MLP 16 input neurons, (corresponding to each joint moment), a hidden layer, and an output layer containing 4 neurons (TMJ signal class). Back-propagation learning, 5000 iterations.	The classification of TMJ sounds based on recorded signals agreed to some extent with physician examination methods (palpation and auscultation). The accelerometer recordings provided additional detail in crepitation signals not detected by physical examination. In patients with lateral cross-bite, 6 out of 11 showed a decrease in TMJ vibrations after treatment, while 2 patients had increased vibrations and 5 showed no change. In Class II Division I patients, 6 out of 21 showed a decrease in vibrations, 17 had increased vibrations, and 5 showed no change after treatment. More patients demonstrated increased TMJ vibrations in the Class II Division I group, and decreased vibrations in the lateral cross-bite group.	Sound
[70]	Soundscoming from the TMJs during opening/closing motion	Nearest linear combination (NLC), Nearest neighbor (NN), and Nearest constrained linear combination (NCLC) were used on either only time-shift, or both time-shift and scale-invariant representations of the signal Reduced Interference distribution (RID)’s	The automated analysis techniques yielded classification outcomes comparable to the previous manual sound classification. Excluding scale invariance significantly enhanced the performance of the classifier. Scale invariance was found to interfere with the frequency content of the signal	Sound
[71]	Set of simulated data was generated and tested by the algorithm. Then TMD data were used. 14 subjects (4 with TMD, 10 healthy) Each subject was captured performing four cycles of chewing motion using color video camera. 400 video frames were obtained per subject.	Singular spectrum analysis	It was interpreted that the SSA technique can be successfully used as a detection method, or at least for extraction of the main signal.	Sound
[72]	26 patients (21 female, 5 males. Aged between 21 and 57.) with 44 TMJs (25 left, 9 right). Patients were used Anterior Repositioning Splint (ARS) for 6 weeks 3 classes (disc displacement (19), disc dislocation with reduction (4), acute disc dislocation without reduction (3))	Evolutionary spectral analysis Parameters (sound type, amplitude, duration and energy) were evaluated	The results showed that 6-week ARS usage reduced amplitude and energy parameters of TMJ sounds	Sound
[73]	35 type 1 (0 to 600 Hz), 31 type 2 (600 to 1200 Hz) and 38 type 3 (above 1200 Hz) TMJ clicks	The Nearest Neighbor (NN), Nearest Linear Combination (NLC) and Nearest Constrained Linear Combination (NCLC)	Linear combinations improved classification of the Reduced Interference Distributions (RID)’s of TMJ sound.	Sound

## Data Availability

Data sharing not applicable.
